# A latent metabolic dysfunction phenotype integrating insulin resistance and leptin is associated with disease severity in COPD

**DOI:** 10.3389/fmed.2026.1915198

**Published:** 2026-10-02

**Authors:** Oana Maria Catana, Denisa Maria Mitroi, Ramona Cioboata, Ovidiu Mircea Zlatian, Mircea Popescu-Driga, Cristina Calarasu, Costin Teodor Streba

**Affiliations:** 1 Doctoral School, University of Medicine and Pharmacy of Craiova, Craiova, Romania; 2 Pneumology Department, University of Medicine and Pharmacy of Craiova, Craiova, Romania; 3 Microbiology Department, University of Medicine and Pharmacy of Craiova, Craiova, Romania; 4 Immunology Department, University of Medicine and Pharmacy of Craiova, Craiova, Romania; 5 Centre for Research in Gastroenterology and Hepatology (CCGH), Craiova, Romania

**Keywords:** chronic obstructive pulmonary disease, disease severity, exacerbations, HOMA-IR, insulin resistance, leptin, metabolic dysfunction

## Abstract

**Background:**

Chronic obstructive pulmonary disease (COPD) is increasingly recognised as a systemic condition in which metabolic dysfunction may influence disease severity and exacerbation risk. The combined effects of insulin resistance and leptin dysregulation on COPD outcomes remain incompletely defined. This study evaluated their associations with exacerbation burden, airflow limitation and patient-reported outcomes, and assessed the discriminative performance of HOMA-IR and serum leptin for identifying advanced disease.

**Methods:**

A cross-sectional observational study was conducted at a tertiary care centre in southern Romania, including 96 patients with COPD and 24 healthy controls. Fasting insulin and serum leptin were measured, and insulin resistance was assessed using HOMA-IR. Spirometry and patient-reported outcomes (CAT, mMRC and SGRQ-C) were assessed in COPD patients. Metabolic markers were analysed individually and as a latent metabolic dysfunction phenotype using generalized structural equation modelling (GSEM).

**Results:**

Compared with controls, COPD patients had higher HOMA-IR (+206.9%), insulin (+112.2%) and leptin (+52.8%) levels. Both HOMA-IR and leptin were inversely correlated with spirometric indices, with stronger associations observed for HOMA-IR. Within the COPD cohort, neither individual metabolic markers nor a latent metabolic dysfunction phenotype integrating HOMA-IR, body mass index and leptin was associated with annual exacerbation rate (latent factor IRR = 1.089, 95% CI: 0.841–1.410, *p* = 0.517). HOMA-IR demonstrated better discrimination of advanced disease than leptin (AUC = 0.769 vs. 0.590, *p* < 0.001).

**Conclusions:**

Metabolic dysfunction is substantially more pronounced in COPD than in individuals without airflow obstruction and correlates with the degree of airflow limitation, but does not independently predict exacerbation frequency within the COPD population. HOMA-IR outperformed leptin as a marker of disease severity, a finding that requires further validation.

## 1 Introduction

Chronic obstructive pulmonary disease (COPD) is now widely recognised as a multidimensional condition whose clinical manifestations extend beyond the paradigm of persistent airflow obstruction (1). From an epidemiological standpoint, COPD imposes a substantial global health burden, with prevalence estimates approaching 480 million cases and mortality figures approximating 3.23 million deaths in 2019, rendering it the third leading cause of death worldwide (2, 3). Projections suggest that COPD cases may approach 600 million by 2050, driven by population ageing, continued tobacco use and exposure to biomass fuels, particularly in low- and middle-income countries (3). However, the consequences of COPD extend well beyond the lungs.

The spirometric threshold formally defining COPD—a post-bronchodilator FEV_1_/FVC ratio of less than 0.70 (4)—has long been acknowledged as an inadequate surrogate of disease severity, given its modest correlation with symptom burden, exercise intolerance, health-related quality of life, and all-cause mortality across affected individuals (5). This discrepancy has shifted attention towards the systemic manifestations of the disease, among which metabolic dysfunction is one of the most clinically relevant but incompletely understood components.

Acute exacerbations constitute a further and equally consequential dimension of COPD morbidity. Defined as episodic deteriorations in respiratory status that accelerate lung function decline and confer a substantial risk of hospitalisation and death, exacerbations are now recognised as the principal modifiable determinant of disease progression and long-term mortality (6). Their occurrence is also influenced by systemic factors, including inflammation, metabolic dysfunction and comorbid disease burden (7). As these processes are poorly captured by spirometric assessment alone, they represent important targets for the identification of clinically relevant biomarkers.

Contemporary understanding of adipose tissue has evolved past its traditional role as an energy store, recognising it as an endocrine organ that regulates immune function, insulin sensitivity and systemic inflammatory signalling (8). This understanding has reshaped how metabolic markers are interpreted in chronic disease. Two mediators are central to the current reorientation in COPD research: leptin, the principal adipokine regulating energy balance and adipose mass, and insulin, the master regulator of glucose homeostasis (9).

Leptin links adipose tissue to whole-body energy regulation by modulating food intake, energy expenditure and substrate utilisation according to nutritional status (10). Beyond its role in energy homeostasis and appetite regulation, leptin also influences glucose metabolism through central and peripheral pathways, including insulin sensitivity, hepatic glucose production, and pancreatic *β*-cell function (10, 11). Leptin additionally has important immunomodulatory properties, acting on several immune cell populations and promoting pro-inflammatory responses, including the production of mediators such as TNF-α and IL-6 (12, 13). These functions position leptin at the interface between adiposity, metabolic regulation, and immune responses.

In COPD, leptin homeostasis is frequently disrupted as a consequence of complex interplay between fat mass, systemic inflammation, physical inactivity, and the neuroendocrine dysregulation that accompanies progressive disease (14). Leptin is additionally expressed within the bronchial submucosa and sputum of COPD patients, where it co-localises with activated CD8⁺ T lymphocytes and correlates with local TNF-α and CRP concentrations, indicating a role in airway inflammation that is not explained by adipose mass alone (15, 16). Impaired leptin-mediated energy regulation leads to impaired glucose homeostasis, altered substrate metabolism, progressive changes in body composition, and increased nutritional vulnerability in COPD (9, 17). As a result, leptin is no longer able to maintain its physiological role as a metabolic regulator, either because its levels are insufficient to support anabolic signalling or because resistance at the tissue level renders elevated concentrations ineffective (16, 18).

Insulin is the principal regulator of glucose homeostasis, coordinating glucose uptake, hepatic gluconeogenesis, and substrate metabolism in peripheral tissues according to the nutritional state (19). Impaired tissue responsiveness to insulin results in insulin resistance (IR), a state commonly quantified using the homeostatic model assessment of insulin resistance (HOMA-IR). In COPD, insulin resistance is promoted by chronic inflammation, physical inactivity, hypoxia and corticosteroid exposure, and affects a substantial proportion of patients independently of airflow limitation (20, 21). Subclinical insulin resistance, detectable by HOMA-IR, affects an estimated 30%–50% of stable COPD patients, and overt type 2 diabetes mellitus is present in 15%–25%, with both rates substantially exceeding those reported in age-matched general populations (9, 21). Importantly, insulin resistance in COPD cannot be explained solely by obesity and may represent a potentially modifiable component of disease burden that develops independently of airflow limitation.

Leptin and insulin do not function independently. Their physiological regulation is closely interconnected. Insulin stimulates leptin secretion from adipose depots, while leptin contributes to the maintenance of peripheral insulin sensitivity (10). In COPD, however, this relationship is subverted. Rather than correcting metabolic imbalance, the interaction between the two hormones sustains and compounds it, perpetuating the dysfunction of the other while both lose biological effectiveness. This feedback loop is most evident in patients with obesity or metabolic syndrome, and it continues to worsen irrespective of any change in the degree of airflow limitation (21).

Although leptin dysregulation and insulin resistance have each been investigated in COPD, evidence evaluating their concurrent disturbances within a unified clinical framework remains limited, particularly in populations with relevant metabolic comorbidities and in direct comparison with healthy controls (22, 23). Furthermore, the extent to which integrated metabolic dysfunction contributes to COPD severity and exacerbation burden has not been fully characterised. Therefore, this study evaluated serum leptin concentrations, fasting insulin levels and insulin resistance (assessed by HOMA-IR) in patients with COPD and healthy controls, and examined their associations with airflow limitation, symptom burden, health-related quality of life and exacerbation frequency. In addition, we explored whether a latent metabolic dysfunction phenotype integrating these abnormalities could provide a more comprehensive representation of the metabolic burden associated with COPD severity and exacerbation risk.

## 2 Materials and methods

### 2.1 Study design and population

A cross-sectional case–control study with retrospective collection of exacerbation history was conducted to investigate the relationship between metabolic markers and clinical outcomes in patients with established COPD and healthy controls.

The study enrolled 120 participants(81 men and 39 women), including 96 patients with COPD admitted to the Department of Pneumology at “Victor Babes” Clinical Hospital of Infectious Diseases and Pneumophthisiology in Craiova, Romania, and 24 non-COPD healthy controls recruited from the same geographic area. The COPD group also included individuals with common metabolic and cardiovascular comorbidities, such as hypertension, obesity, type 2 diabetes, heart failure, obstructive sleep apnoea syndrome, and atrial fibrillation. All participants were enrolled between January and December 2025.

The study was conducted in accordance with the guidelines of the Declaration of Helsinki and approved by the Institutional Review Board. Ethical approval was granted by the University of Medicine and Pharmacy of Craiova (No. 407/20.11.2024) and the “Victor Babes” Clinical Hospital of Infectious Diseases and Pneumophthisiology Craiova, Romania (No. 15524/30.10.2024). Participants were enrolled after providing voluntary informed consent, with no political, social, or religious discrimination, and in strict adherence to data protection legislation.

### 2.2 Inclusion and exclusion criteria

Eligibility for the COPD group required: age between 40 and 80 years, confirmed COPD diagnosis based on post-bronchodilator FEV_1_/FVC < 0.70 (in accordance with the 2025 GOLD criteria) and written informed consent. Both ever-smokers and never-smokers were eligible, reflecting the contribution of non-tobacco exposures to COPD in this region. We did not exclude patients with metabolic and cardiovascular comorbidities, including type 2 diabetes mellitus, arterial hypertension, heart failure, obesity, obstructive sleep apnoea syndrome and atrial fibrillation. Their inclusion reflects the high prevalence of these conditions in COPD and allows assessment of their independent influence on the leptin–insulin metabolic axis. A history of treated pulmonary tuberculosis was not exclusionary, given its high regional prevalence and severity in our population (24). Control participants had no history of chronic respiratory disease, no active respiratory symptoms, and no major comorbidities. We confirmed the absence of airflow obstruction in all controls by a post-bronchodilator FEV_1_/FVC ratio of 0.70 or above on spirometry.

Exclusion criteria for all participants included age <40 years or >80 years, established diagnosis of pulmonary disease other than COPD, active malignancy, severe hepatic or renal disease, use of immunomodulatory therapy or systemic corticosteroids for more than four weeks in the six months preceding enrolment, acute exacerbation of COPD in the four weeks before enrolment, and refusal to provide informed consent. We excluded patients with severe hepatic or renal disease due to their independent effects on circulating leptin and insulin levels (25, 26). Long-term corticosteroid users were also excluded owing to the well-established effects of systemic glucocorticoids on insulin sensitivity (27). Participants with incomplete data were not included in the final analysis.

### 2.3 COPD diagnosis, severity classification and exacerbation history

We confirmed an existing COPD diagnosis at enrolment in accordance with the 2025 GOLD criteria (4), based on documented chronic respiratory symptoms, relevant exposure history, and spirometric evidence of persistent airflow limitation. A post-bronchodilator FEV_1_/FVC ratio below 0.70 was required. Disease severity was classified according to GOLD spirometric stages using the percentage of predicted FEV_1_: Stage I (≥80%), Stage II (50%–79%), Stage III (30%–49%) and Stage IV (<30%). Clinical group assignment (Group A, B, or E) was determined on the basis of symptom burden, assessed by the COPD Assessment Test (CAT) and the modified Medical Research Council dyspnoea scale (mMRC), combined with exacerbation history in the preceding twelve months, in accordance with the GOLD 2025 ABE classification (4).

Exacerbation data were obtained retrospectively from medical records and patient interviews. An exacerbation was defined as an acute worsening of respiratory symptoms beyond normal day-to-day variation that required a change in pharmacological treatment. For each patient, we recorded the total number of exacerbations over the preceding twelve months, the time from COPD diagnosis to the first exacerbation, and the resulting annual exacerbation rate. Patients were classified as frequent exacerbators if they reported more than two exacerbations in the preceding twelve months. This threshold is more stringent than the GOLD criterion of two or more moderate exacerbations per year, and was adopted because the latter would have classified 95.8% of this tertiary-care cohort as frequent exacerbators, leaving an uninformative reference group.

### 2.4 Clinical and anthropometric data

From medical records and direct patient assessment, we collected demographic data, including age, sex, and place of residence (urban or rural). Clinical history included age at COPD diagnosis, smoking status age at smoking initiation and total years of smoking. Smoking status was recorded as current smoking at the time of enrolment. Patients who had previously smoked but had ceased before enrolment and those who had never smoked were classified as non-current smokers. Cumulative exposure was captured separately as total years of smoking.

Details of each patient's pharmacological treatment regimen (LABA monotherapy, LABA/LAMA, ICS/LABA or ICS/LABA/LAMA therapy) and use of long-term oxygen therapy (LTOT) were recorded. The documented comorbidities were type 2 diabetes, hypertension, heart failure, atrial fibrillation, obesity and obstructive sleep apnoea syndrome (OSAS). History of pulmonary tuberculosis was also noted for each participant. Body mass index (BMI) was calculated as weight in kilograms divided by the square of height in meters and categorized as underweight (<18.5 kg/m^2^), normal weight (18.5–24.9 kg/m^2^), overweight (25.0–29.9 kg/m^2^), and obese (≥30.0 kg/m^2^).

### 2.5 Symptom assessment and patient-reported outcomes

Symptom burden and health-related quality of life were assessed using three validated patient-reported outcome instruments completed at the time of enrolment. The COPD Assessment Test (CAT) is an eight-item questionnaire that measures the impact of COPD on health status, with scores ranging from 0 to 40; higher scores reflect greater disease burden (28). The modified Medical Research Council dyspnoea scale (mMRC) grades breathlessness severity on a five-point scale from 0 (breathlessness only with strenuous exercise) to 4 (too breathless to leave the house or dress and undress) (29). The St George's Respiratory Questionnaire for COPD (SGRQ-C) measures disease-specific health status on a scale from 0 to 100, with higher scores indicating poorer quality of life (30). All three instruments are well validated in COPD and are widely used in both clinical practice and research.

### 2.6 Spirometry

Pulmonary function testing was performed according to the American Thoracic Society/European Respiratory Society (ATS/ERS) standardised recommendations (31) using a Spirolab IV spirometer (Medical International Research, Rome, Italy). For each participant, we recorded absolute and percentage predicted values for forced expiratory volume in one second (FEV_1_), forced vital capacity (FVC), forced expiratory flow between 25% and 75% of FVC (FEF_25%–75%_), and the FEV_1_/FVC ratio (Tiffeneau index). Predicted values were derived from reference equations adjusted for age, sex, height and ethnicity. Spirometry was performed during a stable clinical state, characterised by the absence of acute respiratory symptoms, haemodynamic instability, or other conditions that could compromise test performance and interpretation.

### 2.7 Laboratory measurements

Venous blood samples(6 mL per patient) were collected in the morning after an overnight fast of at least 8 h using serum separator tubes for biochemical analyses and EDTA tubes for haematological testing. Serum was separated by centrifugation at 3,000 rpm for 10 min at room temperature and stored at −20°C until analysis. Routine haematological and biochemical investigations, including complete blood count, fasting glucose, C-reactive protein (CRP), fibrinogen and erythrocyte sedimentation rate (ESR), were performed using standard automated laboratory methods.

Serum leptin and insulin were measured at the Department of Immunology, University of Medicine and Pharmacy of Craiova, using Quantikine ELISA Kits (Leptin, DLP00; Insulin, DINS00; R&D Systems, Bio-Techne Corporation, Minneapolis, MN, USA), according to the manufacturer's instructions. We calculated insulin resistance using the homeostatic model assessment of insulin resistance (HOMA-IR), with the formula: fasting insulin (µIU/mL) × fasting glucose (mg/dL)/405.

We defined hyperinsulinaemia as a fasting serum insulin concentration > 25 µIU/mL, corresponding to the upper limit of the standard clinical reference range (2.6–24.9 µIU/mL) reported by major reference laboratories (32) and used by the performing laboratory. This threshold identifies overt clinical hyperinsulinaemia, complementing the continuous HOMA-IR measure used for assessing the broader spectrum of insulin resistance throughout the analyses. Given the marked sex-related differences in circulating leptin concentrations (11, 33), elevated leptin levels were defined using the sex-specific upper reference limits reported by the assay manufacturer (>11.1 ng/mL for men and >77.3 ng/mL for women), derived from apparently healthy volunteers.

### 2.8 Statistical analysis

Continuous variables were summarised as mean ± standard deviation (SD), while categorical variables were presented as absolute (n) and relative (%) frequencies. Normality was assessed prior to selection of appropriate statistical tests. Between-group comparisons were performed using the independent samples t-test for normally distributed variables and the Mann–Whitney *U*-test for non-normally distributed variables. Categorical variables were compared using the chi-square test or Fisher's exact test, as appropriate. Risk ratios (RR) were calculated for categorical variables, and effect sizes for continuous variables were expressed as Cohen's d with 95% confidence intervals.

Because COPD patients and controls differed substantially in age and were not individually matched, between-group comparisons of metabolic markers were repeated using multivariable linear regression on log-transformed values (HOMA-IR, insulin, leptin), adjusting for age, sex, body mass index and smoking duration. Adjusted between-group differences are reported as percentage differences in geometric means, derived from exponentiated regression coefficients, with 95% confidence intervals.

Spearman's rank correlation was used to assess the associations between serum leptin, HOMA-IR and spirometric indices. Lung function parameters were additionally compared between patients with frequent exacerbations (>2 per year) and those with fewer exacerbations (≤2 per year) using the Mann–Whitney *U*-test. Patient-reported outcomes (CAT score, mMRC dyspnoea grade, and SGRQ-C score) were summarised according to insulin and leptin status.

Metabolic subgroups were defined across the full study sample and therefore include control participants in the normal-insulin and normal-leptin groups; these comparisons are descriptive. A sensitivity analysis restricted to COPD patients was performed using the Mann–Whitney *U*-test. Observed differences were considered in relation to established minimal clinically important differences (MCIDs), which serve as reference thresholds for interpreting the magnitude of change in these instruments.

To evaluate whether hyperinsulinaemia independently predicts frequent exacerbations, we used propensity score matching (PSM) with a Gaussian kernel and restricted the analysis to the common support region. Propensity scores were estimated using a probit regression model including nonlinear squared terms for age and BMI, their interaction term (BMI × age), sex and smoking duration. Covariate balance between matched groups was confirmed using standardised mean differences, with values < 0.10 indicating adequate balance. The average treatment effect on the treated (ATT) was reported with its standard error and t-statistic.

Exacerbation frequency was analysed using negative binomial regression. The model included metabolic markers (log-transformed HOMA-IR, centred BMI, log-transformed leptin), demographic variables (age, sex, residence), comorbidities (obesity, OSAS, diabetes mellitus, hypertension, heart failure, atrial fibrillation), exposure history (smoking status and duration, tuberculosis history), GOLD stage, and treatment regimen. Results were reported as incidence rate ratios (IRR) with 95% confidence intervals. Covariates were selected *a priori* on the basis of established associations with COPD exacerbation frequency. Variance inflation factors were examined to identify redundancy among predictors, and variables showing substantial collinearity with the metabolic exposures of interest were excluded from the primary model.

To evaluate the combined contribution of metabolic abnormalities to exacerbation burden, a generalised structural equation model (GSEM) with a Poisson distribution, log link, and robust standard errors was fitted on the full study sample to test whether a latent metabolic dysfunction factor was associated with annual exacerbation rate. The latent factor was built around HOMA-IR as the anchor indicator, with centred BMI and log-transformed serum leptin as additional indicators. The structural pathway was adjusted for age, sex, and smoking duration, with annual exacerbation rate as the outcome variable.

The generalised structural equation model was additionally repeated restricted to COPD patients only (*n* = 96), since healthy controls have no clinically meaningful exacerbation outcome and were assigned an exacerbation count of zero in the full-sample model. The COPD-only model is presented as the primary GSEM analysis; the full-sample model (*n* = 120) is retained as a sensitivity analysis.

Receiver operating characteristic (ROC) analysis was performed to evaluate the ability of HOMA-IR and serum leptin to discriminate between patients with advanced COPD (GOLD stages III–IV) and those with milder disease (GOLD stages I–II). Area under the curve (AUC) values with 95% confidence intervals were reported. Differences in discriminative performance between the two markers were assessed using the chi-square statistic generated by ROC comparison analysis.

All analyses were conducted using Stata/SE 17.0 (StataCorp LLC, College Station, TX, USA). Statistical significance was defined as a two-sided *p*-value < 0.05.

## 3 Results

### 3.1 Baseline characteristics of the study population

Before examining the relationships between metabolic markers and clinical outcomes, we characterised the two study groups in order to establish the extent of metabolic divergence between them and to identify demographic and clinical factors that might influence subsequent comparisons.

A total of 120 participants were enrolled: 96 with confirmed COPD diagnosis and 24 non-COPD healthy controls. The two groups did not differ significantly in sex distribution (69.79% male in COPD vs. 58.33% in controls, RR = 1.20, *p* = 0.407) or smoking prevalence (56.25% vs. 45.83%, RR = 1.22, *p* = 0.359), suggesting that between-group differences in metabolic parameters were not primarily attributable to these characteristics. Detailed demographic, clinical and metabolic data are presented in Table 1.

**Table 1:** Demographic, clinical, and metabolic characteristics in COPD patients (*n* = 96) and healthy controls (*n* = 24). Data presented as mean ± SD or *n* (%). Effect sizes are expressed as Cohen's d with 95% confidence intervals for continuous variables and risk ratios (RR) for categorical variables.

Characteristic	Healthy controls (*n* = 24)	COPD (*n* = 96)	RR/Cohen's d	*p*- value	Total
Demographics					
Male sex, *n* (%)	14 (58.33%)	67 (69.79%)	1.20	0.407	67.50%
Age (years)	51.12 ± 8.73	66.06 ± 9.18	1.37	<0.001	63.07
Smoking history					
Current smoker, *n* (%)	11 (45.83%)	54 (56.25%)	1.22	0.359	54.17%
Smoking duration (years)	22.00 ± 11.48	36.88 ± 13.37 (+67.65%)	0.49	<0.001	34.08
Age at smoking onset (years)	23.12 ± 7.88	26.68 ± 8.68 (+15.37%)	0.41	0.137	26.01
Tuberculosis history, *n* (%)	0 (0.00%)	12 (12.50%)	—	0.067	10.00%
Anthropometric					
BMI (kg/m^2^)	24.90 ± 2.99	27.24 ± 7.31 (+9.39%)	0.34	0.128	26.77
Comorbidities					
Obesity, *n* (%)	0 (0.00%)	36 (37.50%)	—	<0.001	30.00%
Hypertension, *n* (%)	0 (0.00%)	66 (68.75%)	—	<0.001	55.00%
Heart failure, *n* (%)	0 (0.00%)	27 (28.13%)	—	0.003	22.50%
Atrial fibrillation, *n* (%)	0 (0.00%)	14 (14.58%)	—	0.046	11.67%
Type 2 diabetes, *n* (%)	0 (0.00%)	30 (31.25%)	—	0.001	25.00%
OSAS, *n* (%)	0 (0.00%)	20 (20.83%)	—	0.014	16.67%
Metabolic markers					
Leptin (ng/mL)	25.51 ± 8.03	38.97 ± 14.19 (+52.77%, adjusted* +36.0%)	1.02	<0.001	36.28
Insulin (*μ*IU/mL)	13.57 ± 3.06	28.80 ± 9.27 (+112.21%, adjusted* +78.6%)	1.81	<0.001	25.75
HOMA-IR	2.78 ± 0.76	8.52 ± 4.65 (+206.92%, adjusted* +131.7%)	1.37	<0.001	7.37

* Adjusted for age, sex, BMI and smoking duration. BMI, body mass index; HOMA-IR, homeostatic model assessment of insulin resistance; OSAS, obstructive sleep apnoea syndrome.

Patients with COPD were significantly older than controls (66.06 ± 9.18 vs. 51.12 ± 8.73 years, *p* < 0.001), with a mean age difference of nearly 15 years. Smoking duration was also longer in COPD patients (36.88 ± 13.37 vs. 22.00 ± 11.48 years of smoking, *p* < 0.001), consistent with the dose-dependent relationship between cumulative tobacco exposure and airflow obstruction. Age at smoking initiation did not differ significantly between groups (26.68 ± 8.68 vs. 23.12 ± 7.88 years, *p* = 0.137).

As expected given the eligibility criteria, none of the healthy controls presented with any of the recorded comorbidities, in contrast to the COPD group where hypertension was present in 68.75% of patients (*p* < 0.001), followed by obesity (37.50%, *p* < 0.001), type 2 diabetes (31.25%, *p* < 0.001), heart failure (28.13%, *p* = 0.003), obstructive sleep apnoea syndrome (20.83%, *p* = 0.014), and atrial fibrillation (14.58%, *p* = 0.046). History of pulmonary tuberculosis was recorded in 12.50% of COPD patients (*p* = 0.067), and BMI was numerically higher in the COPD group (27.24 ± 7.31 vs. 24.90 ± 2.99 kg/m^2^; *p* = 0.128), though neither difference reached statistical significance.

The most substantial differences between groups were identified in the metabolic markers. Serum insulin levels in patients with COPD were more than twice those observed in controls (28.80 ± 9.27 vs. 13.57 ± 3.06 µIU/mL, *p* < 0.001; Cohen's d = 1.81, 95% CI: 1.30–2.31). HOMA-IR was also significantly elevated in the COPD group (8.52 ± 4.65 vs. 2.78 ± 0.76, *p* < 0.001; Cohen's d = 1.37, 95% CI: 0.89–1.85), corresponding to a 206.92% relative increase. Serum leptin concentrations were 52.77% higher in COPD patients than in controls (38.97 ± 14.19 vs. 25.51 ± 8.03 ng/mL, *p* < 0.001; Cohen's d = 1.02, 95% CI: 0.55–1.48). All three metabolic markers showed large, unadjusted effect sizes.

Because the COPD and control groups differed substantially in age (66.06 vs. 51.12 years) and were not individually matched, these unadjusted comparisons should not be interpreted as reflecting an effect of COPD independent of age, adiposity and smoking exposure. After adjustment for age, sex, body mass index and smoking duration, between-group differences in metabolic markers were attenuated but remained substantial and highly significant: HOMA-IR was 131.7% higher (95% CI: 87.3–186.7%, *p* < 0.001), fasting insulin was 78.6% higher (95% CI: 57.1–103.2%, *p* < 0.001), and serum leptin was 36.0% higher (95% CI: 25.0–48.0%, *p* < 0.001) in patients with COPD. Age was not independently associated with HOMA-IR or insulin (*p* = 0.123 and 0.051) and only weakly associated with leptin (*p* = 0.037), whereas body mass index was the dominant covariate in all three models (*p* < 0.001), indicating that the age difference between groups was not the principal driver of the observed metabolic differences.

The magnitude of these metabolic differences, particularly the threefold difference in HOMA-IR, establishes the contrast that motivates the analyses reported below. At the same time, the groups differed substantially in age, cumulative tobacco exposure and comorbidity burden, and age, sex, body mass index and smoking duration were therefore included as covariates in the multivariable analyses. The distribution of key continuous variables across both groups is illustrated in Figure 1.

**Figure 1:**
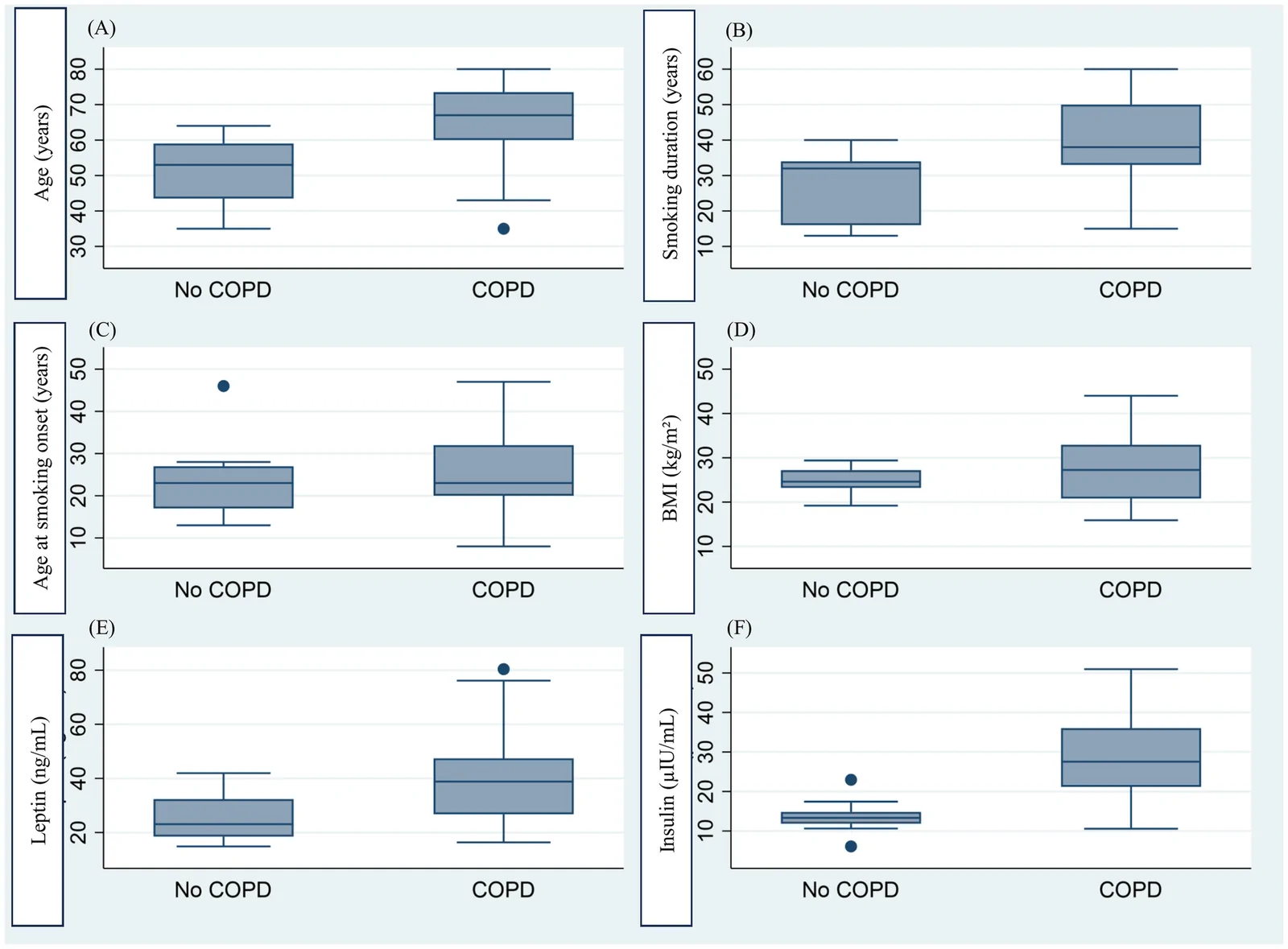
Boxplot distributions of selected demographic and metabolic variables in COPD patients (*n* = 96) and healthy controls (*n* = 24). Panels **(A–F)** show: age **(A)**, smoking duration **(B)**, age at smoking onset **(C)**, body mass index **(D)**, serum leptin concentration **(E)**, and serum insulin concentration **(F)**. Between-group comparisons (Mann–Whitney *U*-test): **(A)** *p* < 0.001; **(B)** *p* < 0.001; **(C)** *p* = 0.134; **(D)** *p* = 0.185; **(E)** *p* < 0.001; **(F)** *p* < 0.001. BMI, body mass index.

### 3.2 Metabolic markers, lung function and exacerbation burden

Having established that metabolic markers were markedly elevated in COPD, we next examined whether the degree of metabolic disturbance was related to the severity of airflow limitation and to exacerbation burden within the COPD cohort itself. This distinction is important, as an association across groups does not necessarily imply a gradient within the diseased population.

HOMA-IR demonstrated moderate inverse correlations with spirometric parameters, with the strongest associations observed for FEF_25%–75%_ (*r* = −0.49), FEV_1_ (*r* = −0.46), FEV_1_/FVC ratio (*r* = −0.45) and FVC (*r* = −0.33). Serum leptin concentrations were also negatively correlated with all lung function parameters, although these relationships were weaker than those observed for HOMA-IR, with the strongest identified for: FEF_25%–75%_ (*r* = −0.28), FEV_1_/FVC ratio (*r* = −0.28) and FEV_1_ (*r* = −0.26).

A moderate positive correlation was observed between HOMA-IR and leptin (*r* = 0.65), consistent with a shared metabolic phenotype. All associations between metabolic markers and spirometric indices reached statistical significance (*p* < 0.05), with the exception of the leptin–FVC association (*r* = −0.16, *p* = 0.085). The complete correlation matrix is presented in Table 2.

**Table 2:** Correlations between metabolic markers and spirometric indices in patients with COPD.

Variable	HOMA-IR	Leptin	FEV_1_	FVC	FEV_1_/FVC ratio	FEF_25−75%_
HOMA-IR	1.00					
Leptin	0.65	1.00				
FEV_1_	−0.46	−0.26	1.00			
FVC	−0.33	−0.16*	0.91	1.00		
FEV_1_/FVC ratio	−0.45	−0.28	0.86	0.60	1.00	
FEF_25%−75%_	−0.49	−0.28	0.91	0.78	0.85	1.00

Values represent Spearman correlation coefficients. All correlations were statistically significant (*p* < 0.05), except for the leptin–FVC association. HOMA-IR, homeostatic model assessment of insulin resistance; FEV_1_, forced expiratory volume in one second; FVC, forced vital capacity; FEF_25%–75%_, forced expiratory flow between 25% and 75% of FVC.

The asterisk * indicates that the leptin–FVC correlation was not statistically significant (*r* = −0.16, *p* = 0.085).

Spirometric parameters differed significantly between patients with ≤2 and >2 exacerbations per year (Figure 2). FEV_1_ was lower in the frequent exacerbator group (38.0 ± 16.4% vs. 60.7 ± 22.3%, *p* < 0.001), as were FVC (71.8 ± 20.4% vs. 99.2 ± 24.1%, *p* < 0.001), FEF_25%–75%_ (25.7 ± 14.4% vs. 42.2 ± 20.8%, *p* = 0.001), and the FEV_1_/FVC ratio (51.3 ± 12.9% vs. 58.8 ± 13.2%, *p* = 0.002). Among the spirometric indices, FEF_25%–75%_ exhibited the greatest between-group difference—approximately 39% lower in patients experiencing >2 exacerbations per year.

**Figure 2:**
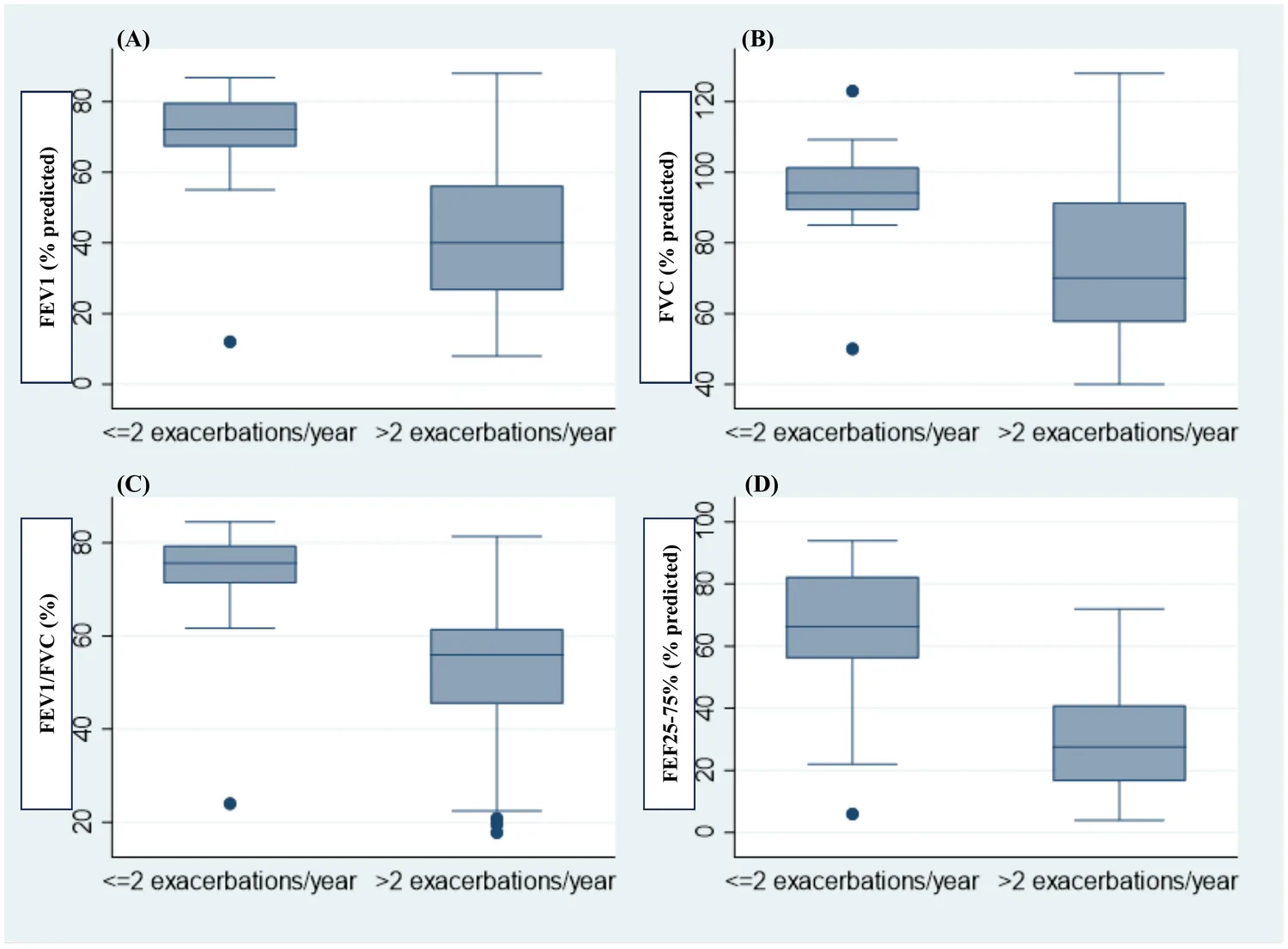
Boxplot distributions of spirometric parameters stratified by exacerbation frequency. Panels **(A–D)** show: FEV_1_ **(A)**, FVC **(B)**, FEV_1_/FVC ratio **(C)**, and FEF_25%–75%_ **(D)** in patients with ≤2 (*n* = 20) and >2 exacerbations (*n* = 76) per year. Between-group comparisons (Mann–Whitney *U*-test): **(A)** *p* < 0.001; **(B)** *p* < 0.001; **(C)** *p* = 0.002; **(D)** *p* = 0.001. FEV_1_, forced expiratory volume in one second; FVC, forced vital capacity; FEF_25%–75%_, forced expiratory flow between 25% and 75% of FVC.

Two patterns emerge from these analyses. First, insulin resistance tracked more closely with airflow limitation than leptin across every spirometric domain, with the strongest association observed for FEF_25%–75%_, an index of small airway function. Second, patients with frequent exacerbations showed substantially poorer lung function throughout, indicating that exacerbation burden in this cohort is closely tied to the degree of functional impairment rather than to metabolic status.

### 3.3 Symptom burden and health status across metabolic subgroups

The CAT and mMRC instruments assess symptom burden and contribute to the GOLD ABE classification, while the SGRQ-C measures health-related quality of life. In this cohort, 4 patients were classified in group A, 17 in group B and 75 in group E.

Patient-reported outcome scores were higher in metabolic subgroups defined by both insulin and leptin status when the full study sample was considered. Among patients with hyperinsulinaemia (*n* = 57), the mean CAT score was 22.75 ± 9.44 compared to 15.68 ± 12.48 in those with normal insulin levels (*n* = 63), a difference of 7.07 points (95% CI: 3.09–11.05). mMRC dyspnoea grade was 0.70 points higher (95% CI: 0.25–1.14) and SGRQ-C was 17.75 points higher (95% CI: 9.37–26.13) in the hyperinsulinaemic subgroup. A similar pattern was observed by leptin status (*n* = 45 hyperleptinaemic vs. *n* = 75 normoleptinaemic): CAT differed by 8.29 points (95% CI: 4.48–12.09), mMRC by 0.83 points (95% CI: 0.39–1.27), and SGRQ-C by 17.65 points (95% CI: 9.44–25.86).

However, these subgroups were defined across the full study sample and therefore include the 24 healthy controls within the “normal insulin” and “normal leptin” categories. Because metabolic status is confounded with COPD status in this comparison, we repeated the analysis restricted to COPD patients only (*n* = 96). In this sensitivity analysis, none of the three patient-reported outcomes differed significantly by insulin or leptin status (all *p* ≥ 0.29, Mann–Whitney *U*-test). For insulin status, the direction of the CAT difference reversed: patients with normal insulin levels had a numerically higher mean CAT score (24.15 ± 7.69) than those with hyperinsulinaemia (22.75 ± 9.44, difference −1.40, 95% CI: −4.89 to 2.09, *p* = 0.322). We therefore conclude that the patient-reported outcome differences reported for the full sample reflect the contrast between COPD patients and healthy controls rather than a gradient of symptom burden within COPD according to metabolic status.

Detailed descriptive results are presented in Table 3 and Figure 3. These analyses describe symptom burden and health status across metabolic subgroups. Exacerbation risk is addressed separately in the following subsections.

**Table 3:** Patient-reported outcomes in metabolic subgroups defined by insulin and leptin status.

Outcome	High insulin (*n* = 57)	Normal insulin (*n* = 63)	Mean difference (95% CI)	*p-*value
CAT	22.75 ± 9.44	15.68 ± 12.48	+7.07 (95% CI 3.09 to 11.05)	*<*0.001
mMRC	2.49 ± 1.12	1.79 ± 1.33	+0.70 (95% CI 0.25 to 1.14)	0.003
SGRQ-C	51.35 ± 18.60	33.60 ± 27.28	+17.75 (95% CI 9.37 to 26.13)	*<*0.001
	High leptin (*n* = 45)	Normal leptin (*n* = 75)	Mean difference (95% CI)	*p*-value
CAT	24.22 ± 8.88	15.93 ± 12.04	+8.29 (95% CI 4.48 to 12.09)	*<*0.001
mMRC	2.64 ± 1.11	1.81 ± 1.28	+0.83 (95% CI 0.39 to 1.27)	*<*0.001
SGRQ-C	53.06 ± 19.15	35.41 ± 26.00	+17.65 (95% CI 9.44 to 25.86)	*<*0.001

Data presented as mean ± SD. Subgroups were defined across the full study sample (*n* = 120). In a sensitivity analysis restricted to COPD patients (*n* = 96), all between-group differences were non-significant (*p* ≥ 0.29). CAT, COPD Assessment Test; mMRC, modified Medical Research Council dyspnoea scale; SGRQ-C, St. George's Respiratory Questionnaire for COPD.

**Figure 3:**
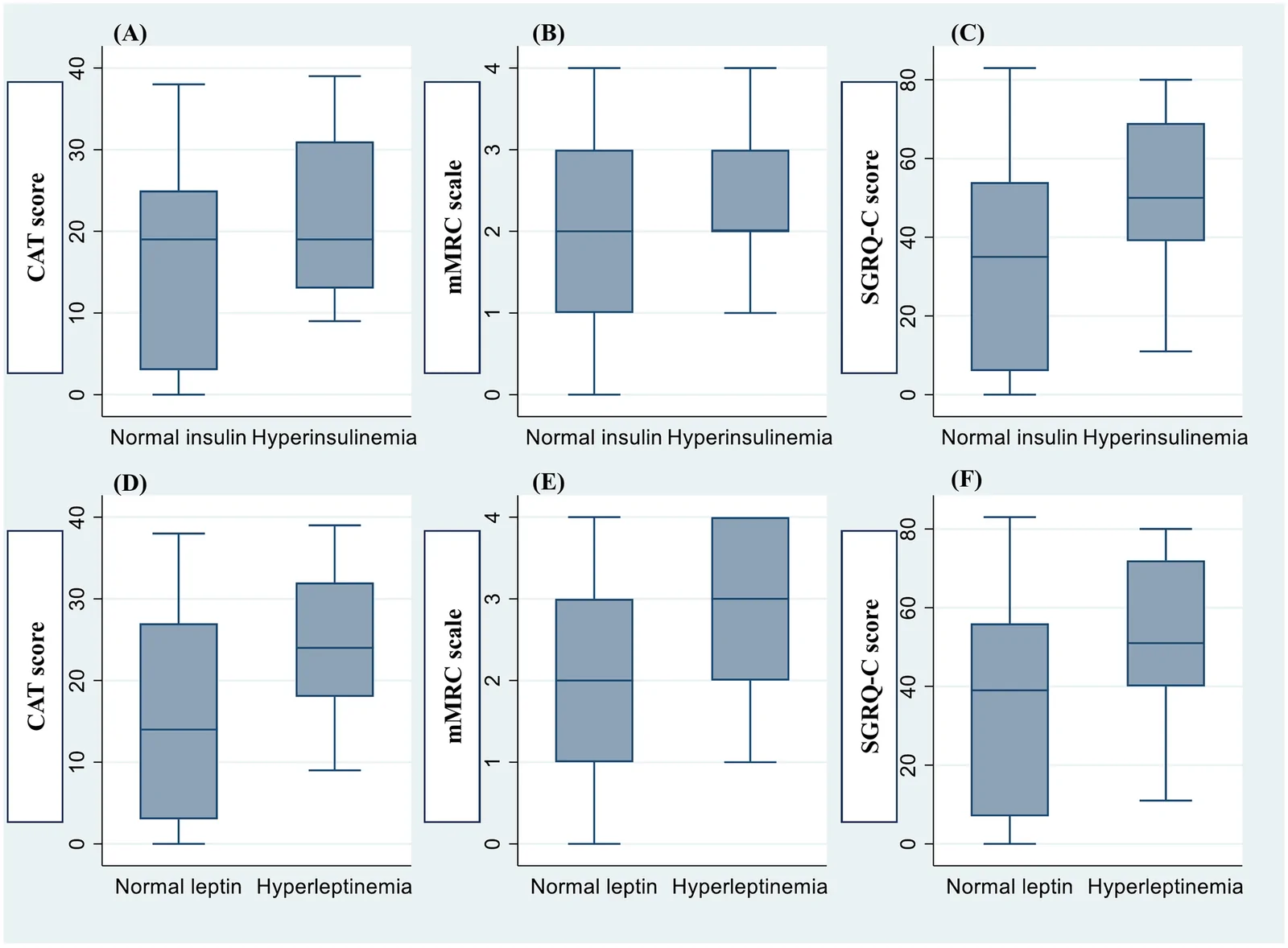
Boxplot distributions of patient-reported outcomes according to insulin and leptin status. Panels **(A,D)** show CAT scores, Panels **(B,E)** show mMRC dyspnoea scale, and Panels **(C,F)** show SGRQ-C scores. Subgroups were defined across the full study sample (*n* = 120): hyperinsulinaemia (*n* = 57), normal insulin (*n* = 63); hyperleptinaemia (*n* = 45), normal leptin (*n* = 75). Distributions are presented descriptively. CAT, COPD Assessment Test; mMRC, modified Medical Research Council dyspnoea scale; SGRQ-C, St. George's Respiratory Questionnaire for COPD.

### 3.4 Independent contribution of hyperinsulinaemia to exacerbation burden

The 96 COPD patients reported a total of 493 exacerbations in the preceding twelve months (mean 5.14 per patient, median 5, range 1–12; variance 6.86, variance-to-mean ratio 1.33), giving approximately 26 events per estimated parameter in the multivariable model.

The correlations reported above establish an association between metabolic markers and disease severity, but they cannot determine whether metabolic disturbance contributes to exacerbation burden independently of the clinical factors with which it coexists. We therefore applied two complementary approaches. The first used propensity score matching with a Gaussian kernel, restricted to the common support region, to balance hyperinsulinaemic and normoinsulinaemic patients on the demographic and anthropometric characteristics that differ systematically between them. Propensity scores were estimated from a probit model incorporating nonlinear squared terms for age and BMI, their interaction (BMI × age), sex, and smoking duration. The probit model predicting hyperinsulinaemia was statistically significant (*p* < 0.001), confirming baseline differences between groups prior to matching.

Among the 96 COPD patients, 57 had hyperinsulinaemia (fasting insulin > 25 µIU/mL) and 39 had normal insulin levels. After imposing common support, 27 of 57 hyperinsulinaemic patients (47.4%) and all 39 normoinsulinaemic patients were retained, yielding a matched sample of 66 patients. The ATT was 0.048 (SE: 0.073; *t* = 0.67, *p* > 0.05). After matching on key confounders, hyperinsulinaemia did not independently predict frequent exacerbations when considered in isolation.

Negative binomial regression analysis performed in the COPD study group identified GOLD stage as a significant predictor of exacerbation frequency (IRR = 1.37, 95% CI: 1.22–1.55, *p* < 0.001), corresponding to a 37% increase in exacerbation rate for each one-stage increase in disease severity. Heart failure was also associated with a higher exacerbation rate (IRR = 1.38, 95% CI: 1.18–1.62, *p* < 0.001), while both hypertension (IRR = 0.73, 95% CI: 0.63–0.85, *p* < 0.001) and obstructive sleep apnoea syndrome (IRR = 0.64, 95% CI: 0.53–0.79, *p* < 0.001) were associated with lower exacerbation rates. These findings were consistent across both matched and unmatched samples. The protective association of hypertension may reflect more intensive cardiovascular monitoring and optimised pharmacological management in this subgroup.

These findings mirror the PSM results—none of the evaluated metabolic markers independently predicted exacerbation frequency in the multivariable model: HOMA-IR (IRR = 1.14, 95% CI: 0.91–1.42, *p* = 0.265), centred BMI (IRR = 1.01, 95% CI: 0.98–1.03, *p* = 0.676) and leptin (IRR = 0.84, 95% CI: 0.56–1.27, *p* = 0.405). Similarly, smoking duration, age, sex, obesity, type 2 diabetes, atrial fibrillation, tuberculosis history, long-term oxygen therapy (LTOT), and both active treatment comparators (LABA/LAMA and ICS/LABA/LAMA vs. LABA monotherapy) similarly did not reach significance, with rural residence approaching borderline significance (IRR = 0.89, 95% CI: 0.78–1.02, *p* = 0.084). Full model results are presented in Supplementary Table S1.

Mean variance inflation factor across predictors in the full model was 4.37. Obesity showed substantial collinearity with BMI, and diabetes mellitus with HOMA-IR; both were therefore removed, together with predictors showing no association with the outcome. None of the three metabolic markers reached significance in the parsimonious model: HOMA-IR (IRR = 1.126, 95% CI: 0.947–1.338, *p* = 0.179), centred BMI (IRR = 1.012, 95% CI: 0.993–1.032, *p* = 0.221), and leptin (IRR = 0.907, 95% CI: 0.597–1.378, *p* = 0.647), consistent with the full model. GOLD stage showed a graded, dose-dependent association with exacerbation rate relative to Stage I (Stage II: IRR = 1.703, *p* = 0.051; Stage III: IRR = 2.070, *p* = 0.005; Stage IV: IRR = 2.794, *p* < 0.001). Hypertension (IRR = 0.741, *p* < 0.001) and obstructive sleep apnoea (IRR = 0.632, *p* < 0.001) retained their inverse association, and heart failure its positive association (IRR = 1.329, *p* < 0.001). The persistence of these associations after removal of the collinear terms indicates that they are not artefacts of model instability.

In the parsimonious model, the estimated dispersion parameter was alpha ≈ 2.37 × 10^−9^ (95% CI: 8.43 × 10^−10^–6.68 × 10^−9^), and the log pseudolikelihood of the negative binomial fit (−184.524) was effectively identical to that of the corresponding Poisson fit, indicating no residual overdispersion once covariates were included. This conditional result should be distinguished from the marginal variance-to-mean ratio of the raw exacerbation counts (1.33), which reflects unconditional overdispersion before adjustment. Robust standard errors were used throughout. The parsimonious model is presented in Table 4.

**Table 4:** Predictors of COPD exacerbation frequency: parsimonious negative binomial regression model (*n* = 96).

Predictor	IRR	Std. err.	*p*-value	95% CI
Metabolic markers				
HOMA-IR (log)	1.126	0.099	0.179	0.947–1.338
BMI (centred)	1.012	0.010	0.221	0.993–1.032
Leptin (log)	0.907	0.193	0.647	0.597–1.378
Demographics				
Age (years)	1.005	0.004	0.207	0.997–1.014
Male sex	1.133	0.130	0.275	0.905–1.418
Smoking duration (years)	1.005	0.002	**0** **.** **002**	1.002–1.008
Disease severity				
GOLD Stage II	1.703	0.464	0.051	0.999–2.903
GOLD Stage III	2.070	0.539	**0** **.** **005**	1.243–3.449
GOLD Stage IV	2.794	0.732	**<0** **.** **001**	1.672–4.668
Comorbidities				
Hypertension	0.741	0.054	**<0** **.** **001**	0.642–0.855
Heart failure	1.329	0.093	**<0** **.** **001**	1.158–1.525
OSAS	0.632	0.062	**<0** **.** **001**	0.521–0.767
Dispersion				
Alpha	2.37 × 10^−9^	1.25 × 10^−9^	—	8.43 × 10^−10^–6.68 × 10^−9^

Results are expressed as incidence rate ratios (IRR) with 95% confidence intervals and robust standard errors. GOLD Stage I is the reference category. Bold *p*-values indicate statistical significance (*p* < 0.05). BMI, body mass index; GOLD, Global Initiative for Chronic Obstructive Lung Disease; HOMA-IR, homeostatic model assessment of insulin resistance; IRR, incidence rate ratio; OSAS, obstructive sleep apnoea syndrome.

The convergence of these methodologically distinct approaches is informative: propensity score matching balances groups on measured confounders before comparison, whereas negative binomial regression adjusts for them within a single model. Both indicate that no individual metabolic marker independently predicts exacerbation frequency once established clinical determinants are taken into account.

### 3.5 Latent metabolic dysfunction as a predictor of exacerbation burden

The lack of significant associations for individual metabolic markers in both the propensity score–matched and regression analyses suggests that single biomarkers may not fully capture the complexity of metabolic dysfunction when considered alongside established clinical predictors. To address this, a generalised structural equation model was fitted to evaluate metabolic dysfunction as a latent construct, combining log-transformed HOMA-IR, centred BMI, and log-transformed serum leptin into a single factor rather than treating them as separate predictors. Log-transformed HOMA-IR served as the anchor indicator (loading fixed to 1.000), with centred BMI (*β* = 11.758, *p* < 0.001) and log-transformed leptin (*β* = 0.836, *p* < 0.001) as further indicators. All three loadings were statistically significant, lending support to the validity of a unified metabolic phenotype.

Restricted to COPD patients (*n* = 96), the model converged after 8 iterations (log pseudolikelihood = −569.05). All three indicators loaded significantly onto the latent metabolic factor: log-transformed HOMA-IR (anchor, loading fixed to 1.000), centred BMI (*β* = 18.591, 95% CI: 13.889–23.293, *p* < 0.001), and log-transformed leptin (*β* = 0.932, 95% CI: 0.692–1.172, *p* < 0.001). However, the latent metabolic factor was not significantly associated with annual exacerbation rate (IRR = 1.089, 95% CI: 0.841–1.410, *p* = 0.517), after adjustment for age, sex and smoking duration. In the full-sample model (*n* = 120), the same association was significant (IRR = 1.696, 95% CI: 1.221–2.171, *p* < 0.001). This discrepancy indicates that the full-sample association was driven predominantly by the contrast between COPD patients and healthy controls, who by construction have zero exacerbations and lower metabolic marker levels—rather than by variation in metabolic dysfunction within the COPD population.

The latent metabolic factor cannot therefore be interpreted as an independent predictor of exacerbation burden within COPD. The full-sample result is retained as a sensitivity analysis, illustrating that metabolic dysfunction tracks COPD status (consistent with the adjusted between-group comparisons reported above), without establishing a within-COPD dose–response relationship with exacerbation frequency. The model converged after eight iterations and the GSEM path diagram is presented in Figure 4.

**Figure 4:**
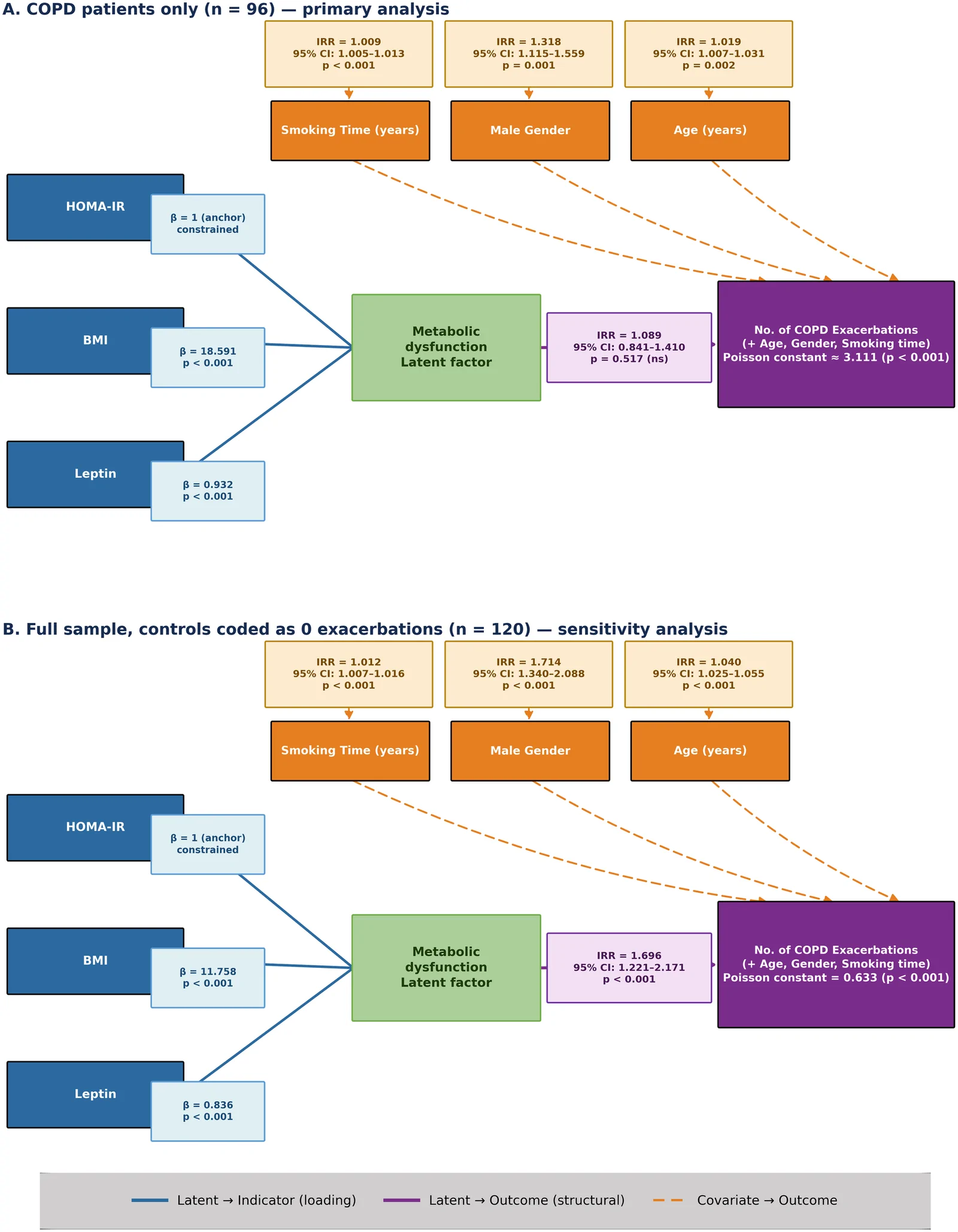
Generalised structural equation model (GSEM) linking latent metabolic dysfunction to COPD exacerbation rate. The latent metabolic dysfunction factor was specified using ln(HOMA-IR), centred BMI, and ln(leptin) as indicators, with ln(HOMA-IR) as the anchor indicator (loading fixed to 1.000). **(A)** Primary analysis restricted to COPD patients (*n* = 96). **(B)** Sensitivity analysis in the full study sample (*n* = 120), in which controls were assigned an exacerbation count of zero. Values shown are unstandardised factor loadings for measurement paths and incidence rate ratios (IRR) with 95% confidence intervals for structural paths. HOMA-IR, homeostatic model assessment of insulin resistance; BMI, body mass index; IRR, incidence rate ratio; CI, confidence interval.

This result is consistent with the analyses reported above, in which the same three markers showed no association when entered separately. Modelling them jointly as a latent construct did not reveal an association with exacerbation rate within the COPD cohort, indicating that the null finding is not attributable to the dilution of individual signals across correlated predictors.

### 3.6 Discriminative performance of HOMA-IR and leptin for disease severity

The preceding analyses examined whether metabolic markers relate to exacerbation burden. We next assessed whether these markers can discriminate patients with advanced airflow limitation from those with milder disease.

ROC analysis revealed a significant difference in discriminative performance between HOMA-IR and leptin for identifying patients with severe-to-very-severe COPD (GOLD III-IV) compared with those with mild-to-moderate disease (GOLD I-II). HOMA-IR demonstrated good discriminative ability, with an AUC of 0.769 (95% CI: 0.682–0.856), indicating that insulin resistance discriminates patients with advanced airflow limitation from those with milder disease.

Leptin, by contrast, showed poor discriminative performance, with an AUC of 0.590 (95% CI: 0.486–0.694), a value only marginally above the 0.50 threshold of no discrimination. The difference between AUCs was statistically significant [*χ*^2^(1) = 21.10, *p* < 0.001], confirming that HOMA-IR is a significantly superior discriminator of COPD severity compared to leptin. ROC curves for both markers are presented in Figure 5.

**Figure 5:**
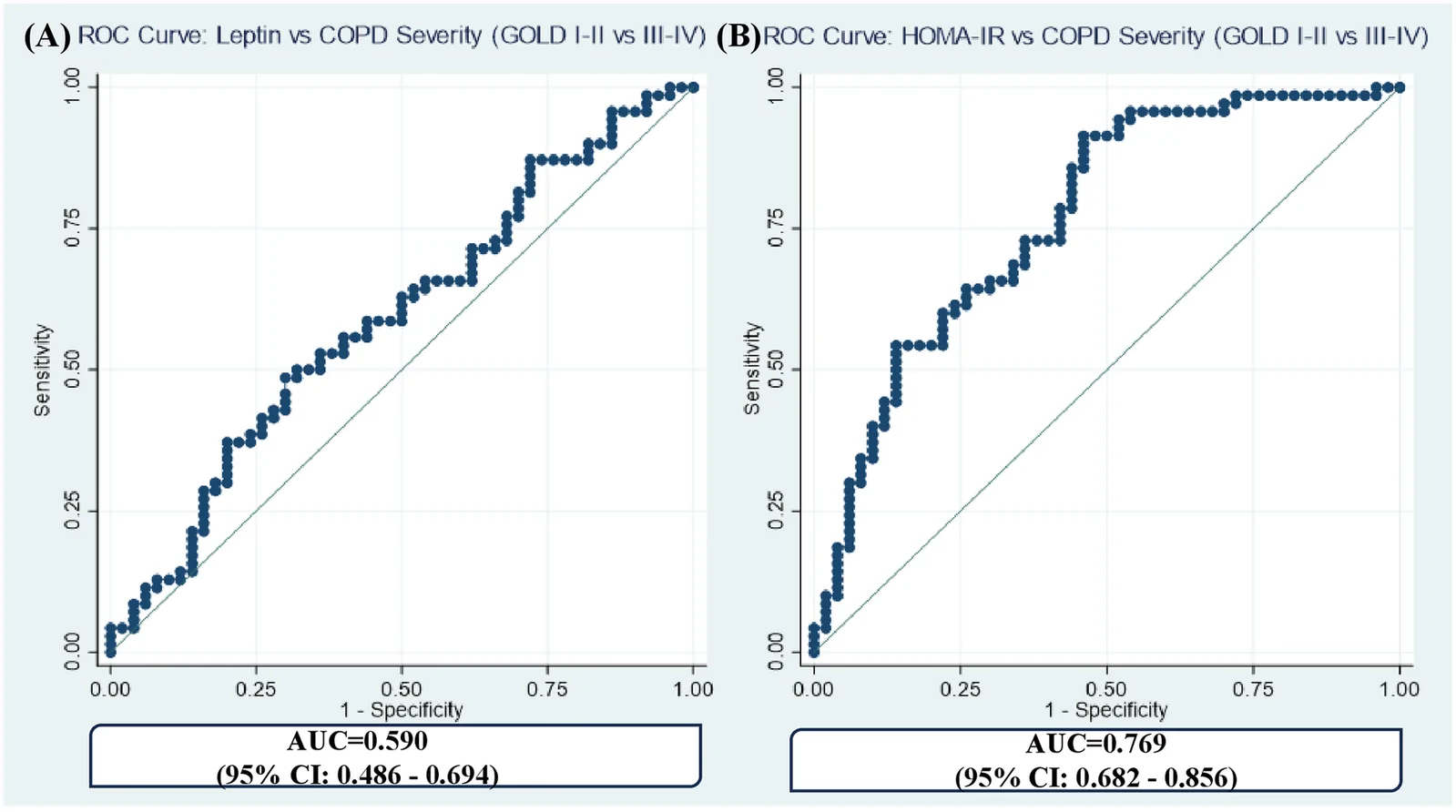
ROC curves of leptin **(A)** and HOMA-IR **(B)** for discrimination of COPD severity. ROC curves showing the ability of leptin **(A)** and HOMA-IR **(B)** to discriminate COPD GOLD I–II from GOLD III–IV. AUC values with 95% confidence intervals are shown below each curve. AUC, area under the curve; HOMA-IR, homeostatic model assessment of insulin resistance.

An overview of the study design and analytical workflow is provided in Figure 6.

**Figure 6:**
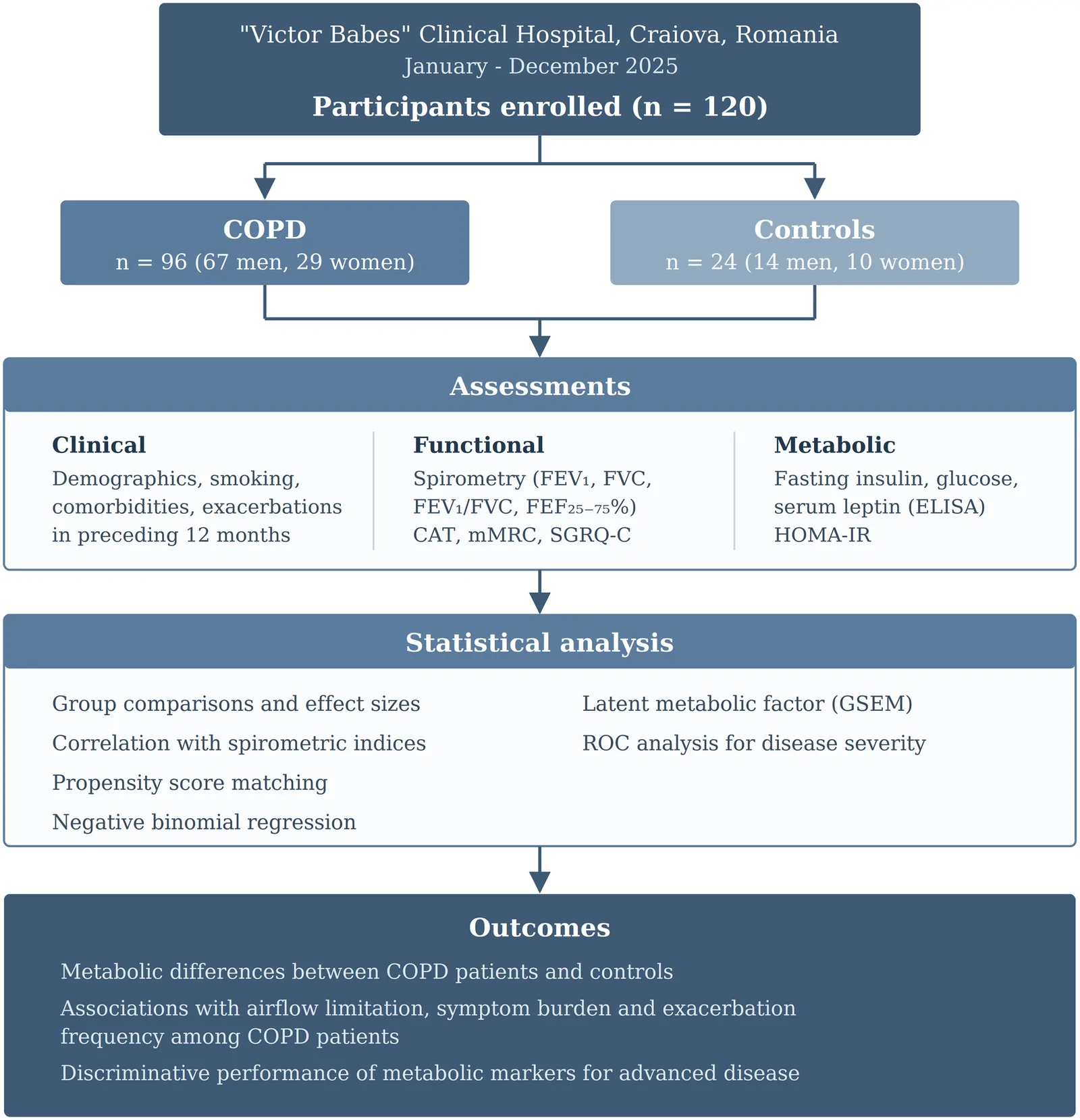
Schematic overview of the study design and analytical workflow. CAT, COPD Assessment Test; ELISA, enzyme-linked immunosorbent assay; FEF_25%–75%_, forced expiratory flow between 25% and 75% of forced vital capacity; FEV_1_, forced expiratory volume in one second; FVC, forced vital capacity; GSEM, generalised structural equation model; HOMA-IR, homeostatic model assessment of insulin resistance; mMRC, modified Medical Research Council dyspnoea scale; ROC, receiver operating characteristic; SGRQ-C, St George's Respiratory Questionnaire for COPD.

## 4 Discussion

This study examined the relationship between metabolic dysfunction characterised by insulin resistance, hyperleptinaemia and their combined expression, and multiple clinical outcomes in COPD. Metabolic dysfunction was markedly more pronounced in COPD than in individuals without airflow obstruction and was associated with the degree of lung function impairment. Within the COPD cohort, however, neither individual markers nor their combined latent expression was associated with exacerbation burden or symptom burden. These results suggest an association between metabolic factors, particularly insulin resistance, and the severity of airflow limitation in COPD.

The demographic and clinical characteristics of the COPD group were consistent with expected epidemiological patterns. Compared with controls, patients with COPD were older, had longer smoking histories and carried a higher comorbidity burden. Sex distribution and smoking prevalence did not differ significantly between groups; however, given the substantial differences in age and comorbidity, the between-group metabolic comparisons were repeated with adjustment for age, sex, body mass index and smoking duration, and the differences remained substantial. Notably, 27 patients (28.1%) had no history of tobacco use, and these were predominantly women (15 of 29 women, compared with 12 of 67 men). This proportion is consistent with the recognised burden of non-smoking COPD in low- and middle-income settings, where biomass fuel combustion and occupational inhalational exposures contribute substantially to disease development, particularly among women (34).

Cardiovascular and metabolic comorbidities were common in the COPD group, particularly hypertension (68.75%), type 2 diabetes (31.25%), and heart failure (28.13%). This pattern reflects the systemic nature of COPD and the shared mechanisms that drive both pulmonary and extrapulmonary disease—oxidative stress, endothelial dysfunction and chronic inflammation (35). These observations are consistent with data from the BOLD and PLATINO multinational cohorts (36, 37), which reported a two- to fourfold higher prevalence of cardiovascular and metabolic comorbidities in COPD patients compared with age-matched controls, independent of smoking status. Recent Romanian evidence further supports this picture, showing that COPD patients carry a particularly high burden of these comorbidities and that prior pulmonary tuberculosis accelerates both the onset and the severity of COPD in this population (24, 34).

The baseline comparison showed clear differences in metabolic markers between groups. HOMA-IR was 206.92% higher in COPD patients than in controls, and fasting insulin was elevated by 112.21%, with large effect sizes, indicating a greater degree of insulin resistance compared with controls. These differences were observed despite similar BMI and obesity prevalence, suggesting that metabolic alterations in COPD are not fully explained by adiposity alone and may involve additional mechanisms such as systemic inflammation, reduced physical activity, corticosteroid exposure, and skeletal muscle dysfunction (38, 39). Serum leptin was 52.77% higher in COPD patients—a disproportionate elevation given the modest and non-significant BMI difference—which may point to adipose tissue inflammation and dysfunction beyond what adiposity alone would predict (15).

Reduced physical activity represents a plausible mediator of the metabolic alterations observed in this study population. Airflow limitation, dynamic hyperinflation and exercise intolerance progressively restrict daily activity in COPD, and physical inactivity independently promotes insulin resistance through reduced skeletal muscle glucose uptake, diminished GLUT4 expression and loss of muscle mass (40). Sedentary behaviour also favours visceral fat accumulation, which in turn raises circulating leptin and sustains low-grade systemic inflammation (9). This creates a self-reinforcing cycle in which metabolic dysfunction and reduced exercise capacity aggravate one another, consistent with the bidirectional relationship between COPD and type 2 diabetes described in recent reviews (21, 41). We did not measure physical activity, exercise capacity or sedentary behaviour, and we therefore cannot determine the extent to which inactivity contributed to the metabolic differences observed; the mMRC score and the activity domain of the SGRQ-C provide only indirect and subjective proxies.

The inverse correlations between insulin resistance, leptin, and spirometric indices indicate a relationship between metabolic dysfunction and airflow limitation in COPD.The associations were generally stronger for HOMA-IR than for leptin across FEV_1_, FVC, TI, and FEF 25%–75%, which is consistent with previous epidemiological studies linking insulin resistance to reduced lung function. In a large population-based cohort, Engström et al. (42) found that elevated fasting insulin was independently associated with lower FEV_1_ and FVC, with the association persisting after adjustment for BMI and smoking status. Hyperinsulinaemia drives airway smooth muscle proliferation through insulin-like growth factor-1 (IGF-1) signalling (43, 44), reduces bronchodilator responsiveness (45), and sustains persistent low-grade systemic inflammation through NF-*κ*B activation (21), all of which may contribute to progressive impairment of lung function in COPD.

The association of both HOMA-IR and leptin with FEF 25%–75% is important, as this parameter reflects small airway function and may detect early changes in airflow limitation. These findings suggest that metabolic dysfunction could have a particular impact on the peripheral airways. Possible mechanisms include airway wall remodelling related to adipose-derived cytokines, increased mucus production linked to leptin signalling in the bronchial epithelium, and the effects of central adiposity on lung mechanics (16, 46).

Although leptin correlated significantly with HOMA-IR (*r* = 0.65) and was inversely associated with all spirometric indices, its correlations with pulmonary function were weaker than those of HOMA-IR. This may reflect leptin's dual role in COPD. At physiological concentrations, leptin exerts pro-inflammatory and bronchoconstrictive effects, but in advanced disease, leptin resistance can uncouple circulating levels from downstream signalling, attenuating its apparent association with functional impairment (16). The relationship between leptin and pulmonary function in COPD is known to be modulated by disease severity and nutritional status, which may explain the heterogeneity reported across previous studies (14).

Sex-related differences in body composition represent an additional source of heterogeneity. Women exhibit substantially higher circulating leptin than men at comparable levels of adiposity, a difference attributable to gonadal steroid regulation and to a greater proportion of subcutaneous fat mass (47). Although sex-specific reference intervals were applied for leptin classification and sex was included as a covariate in all multivariable models, residual sex-related variation may have contributed to the weaker associations observed between leptin and pulmonary function in this cohort. Together, these correlations indicate that metabolic dysfunction is closely linked to the degree of airflow limitation in COPD, although the cross-sectional design does not permit conclusions regarding the direction of this relationship.

The differences observed in CAT, mMRC, and SGRQ-C scores between metabolic subgroups exceeded established MCID thresholds (29, 48, 49) in magnitude. However, these subgroups were defined across the full study sample and therefore differed in COPD status as well as in metabolic status. When the comparison was restricted to COPD patients, no significant differences emerged, indicating that the observed contrast reflects the presence of disease rather than metabolic status *per se*. Symptom burden and health-related quality of life in this cohort therefore appear to be determined primarily by COPD itself, with no detectable additional contribution from insulin or leptin status.

Similar patterns across insulin and leptin subgroups suggest that these markers reflect a shared metabolic phenotype rather than entirely separate pathways. Both hyperinsulinaemia and hyperleptinaemia are features of adipose tissue dysfunction and systemic insulin resistance, conditions linked to chronic low-grade inflammation (12, 13). These inflammatory cascades may worsen dyspnoea, exercise tolerance, and airway inflammation in COPD through pathways not fully reflected in spirometric measures.

Although mMRC scores did not differ significantly between leptin subgroups within the COPD cohort, the biological rationale for a relationship between leptin and dyspnoea perception merits consideration. Leptin receptors are expressed in the hypothalamus, carotid body, and respiratory muscles, and leptin is involved in the regulation of ventilatory drive and respiratory muscle function (50). In COPD, where respiratory muscle fatigue and dynamic hyperinflation already play an important role in dyspnoea, alterations in leptin signalling may further contribute to breathlessness independently of airflow limitation. Vernooy et al. (16) have reported that circulating leptin levels correlate with systemic inflammatory markers, reduced exercise capacity, and poorer quality of life in COPD.

Comparable analyses in the ECLIPSE (7) and COSYCONET (51) cohorts have reported that clustering of metabolic comorbidities is associated with poorer health status regardless of lung function severity. Our data do not reproduce this association within the COPD group, which may reflect the smaller sample size, the high overall symptom burden in a tertiary-care population, or the use of individual biomarkers rather than composite comorbidity clusters.

Propensity score matching showed that hyperinsulinaemia was not independently associated with frequent exacerbations after adjustment for age, BMI, sex, and smoking duration (ATT = 0.048, *p* > 0.05). This finding is consistent with the negative binomial regression analysis, in which HOMA-IR, BMI, and leptin were not significantly associated with exacerbation frequency after accounting for established clinical predictors. Together, these results suggest that individual metabolic markers may not adequately capture the contribution of metabolic dysfunction to exacerbation risk when considered in isolation.

In contrast, GOLD stage showed a graded, dose-dependent association with exacerbation frequency relative to Stage I (Stage II: IRR = 1.703; Stage III: IRR = 2.070; Stage IV: IRR = 2.794), consistent with its established role as a key determinant of disease progression (6). Heart failure was associated with higher exacerbation rates (IRR = 1.329), reflecting the bidirectional cardiopulmonary interaction in COPD (52).

The inverse associations observed for hypertension (IRR = 0.741) and obstructive sleep apnoea (IRR = 0.632) were unexpected. Several explanations are possible, including differences in clinical follow-up between patients with and without recognised cardiovascular or sleep-related comorbidity, residual confounding, or chance findings within a multivariable model. As treatment intensity, CPAP prescription and adherence were not recorded, these associations cannot be interpreted mechanistically and require confirmation in independent cohorts. Notably, they persisted after removal of the collinear terms in the parsimonious model, indicating that they are not artefacts of model instability.

Modelling metabolic dysfunction as a latent construct allowed the shared variance across HOMA-IR, BMI and leptin to be captured in a single factor, avoiding the dilution of individual signals that occurs when correlated markers are entered separately into a regression model. All three indicators loaded significantly onto the latent factor within the COPD cohort, confirming that they measure a coherent underlying construct. However, this factor was not associated with annual exacerbation rate (IRR = 1.089, 95% CI: 0.841–1.410, *p* = 0.517). When the model was fitted to the full study sample, the same association was significant (IRR = 1.696, 95% CI: 1.221–2.171, *p* < 0.001), but this reflects the contrast between COPD patients and healthy controls who by construction contribute no exacerbations and have substantially lower metabolic marker levels, rather than variation in metabolic burden among patients with established disease.

This null result is consistent across three methodologically distinct approaches: propensity score matching, negative binomial regression and latent variable modelling all indicate that metabolic dysfunction does not independently predict exacerbation frequency once established clinical determinants are taken into account. The convergence of these analyses strengthens confidence in the finding. It also suggests that associations reported in some previous COPD studies may partly reflect comparisons with non-COPD reference groups rather than gradients within diseased populations. The latent variable framework nonetheless remains methodologically useful for characterising metabolic phenotypes, and its application to larger COPD cohorts, or to outcomes other than exacerbation frequency, warrants further investigation.

The superiority of HOMA-IR over leptin in discriminating COPD severity has several plausible mechanistic and clinical explanations. HOMA-IR is a composite index of fasting insulin and glucose that captures the degree of systemic insulin resistance. This is closely linked to skeletal muscle dysfunction, systemic inflammation, and oxidative stress, processes that worsen alongside COPD severity and contribute to disease progression independently of airflow limitation (17, 53). The observed AUC of 0.769 reflects good diagnostic performance and is comparable to values reported for established metabolic and inflammatory markers in COPD (54).

An AUC of 0.769 obtained in a single-centre sample of this size indicates discriminative capacity within the derivation dataset only. No internal cross-validation, calibration assessment or external replication was performed, and no optimal threshold was derived. These findings therefore do not establish clinical utility and should be regarded as hypothesis-generating.

In contrast, leptin showed lower discriminative performance (AUC = 0.590). This is biologically coherent, as leptin levels are mostly driven by adipose tissue mass and therefore track more closely with body composition than with airflow limitation. In more advanced COPD stages, weight loss and muscle wasting may lead to lower leptin levels, potentially reducing its ability to distinguish between disease severity (18, 55). By comparison, insulin resistance may persist or increase in these patients, which helps maintain the discriminative signal of HOMA-IR across severity stages (56).

Taken together, these findings suggest that HOMA-IR is more informative than leptin for discriminating disease severity, a distinction that may inform biomarker selection in future research.

The findings of this study have several practical implications. HOMA-IR, a low-cost and widely available laboratory measure, discriminated more severe from milder disease in this cohort and outperformed leptin in this respect, suggesting that it merits evaluation as a candidate severity marker in future studies alongside conventional GOLD staging. Metabolic dysfunction did not, however, relate to exacerbation frequency or symptom burden within the COPD study population, and our data therefore do not support the use of these markers for exacerbation risk stratification. Whether composite metabolic assessment has value in relation to outcomes other than exacerbation frequency, such as functional decline, body composition or mortality, remains to be established.

## 5 Limitations

Several limitations should be considered when interpreting these findings. The study was conducted at a single centre, and its cross-sectional design precludes any inference regarding temporal sequence or causality. The associations reported here cannot establish whether metabolic dysfunction contributes to COPD severity, arises as a consequence of it, or reflects shared upstream determinants. Nevertheless, the consistency of the associations observed across multiple complementary analytical approaches strengthens confidence in the overall findings.

The control group was intentionally selected to be free of major respiratory, metabolic, and cardiovascular comorbidities, and was consequently younger and less exposed to tobacco than the COPD group. This introduces the possibility of healthy-control selection bias, and some of the observed metabolic differences may reflect the combined burden of COPD and its associated comorbidities rather than COPD alone, despite adjustment for relevant covariates in multivariable analyses. Propensity score matching further reduced the effective sample size, and residual confounding by treatment adherence, lifestyle behaviours and socioeconomic conditions cannot be excluded.

Physical activity, exercise capacity, and sedentary behaviour were not assessed. Because physical inactivity may both result from airflow limitation and contribute independently to insulin resistance and adiposity, its potential contribution to the metabolic differences observed in this study could not be quantified. Metabolic assessment was also based exclusively on fasting measurements. Postprandial glucose testing or an oral glucose tolerance test would have provided additional information on glucose tolerance and *β*-cell function beyond that captured by fasting indices. Exacerbation history was reconstructed retrospectively, and biomarkers were measured at enrolment rather than at the time of diagnosis. Analyses of exacerbation timing were therefore not feasible, and exacerbation burden was assessed exclusively through count-based models.

Finally, the structural pathway of the GSEM was adjusted for age, sex and smoking duration only, without accounting for COPD severity, diabetes, cardiovascular comorbidity or medication exposure. While the modelling approach allowed metabolic dysfunction to be examined as a combined construct, further validation in larger, multicentre and more diverse populations is needed.

## 6 Conclusion

This study demonstrates that metabolic dysfunction, characterised by insulin resistance and leptin dysregulation, is markedly more pronounced in patients with COPD compared with healthy controls, together with significant associations between metabolic markers and the degree of airflow limitation. HOMA-IR showed stronger correlations with spirometric indices than leptin across all lung function parameters and discriminated advanced from milder disease with good accuracy, outperforming leptin in this respect. Leptin, whose circulating concentrations depend heavily on body composition and may be uncoupled from downstream signalling in advanced disease, showed weaker discriminative ability.

Within the COPD cohort, however, neither individual metabolic markers nor a composite latent construct was associated with exacerbation frequency or with symptom burden, a null result consistent across propensity-matched, regression and latent variable analyses. These findings indicate that metabolic markers, and HOMA-IR in particular, are associated with the severity of airflow limitation in COPD, but do not support their use for exacerbation risk stratification.

Whether metabolic dysfunction contributes to COPD progression or arises as a consequence of it cannot be determined from a cross-sectional design. Prospective studies in larger and more diverse cohorts are needed to clarify the direction of this relationship and to establish whether metabolic assessment adds clinically useful information beyond spirometry.

## Data Availability

The original contributions presented in the study are included in the article/Supplementary Material, further inquiries can be directed to the corresponding author/s.
