# Supplementary material for: A latent metabolic dysfunction phenotype integrating insulin resistance and leptin is associated with disease severity in COPD
Source: Front Med (Lausanne). 2026 Oct 2;13:1915198. doi: 10.3389/fmed.2026.1915198 (PMC13672520; doi:10.3389/fmed.2026.1915198)
Supplement: Supplementary file 1 [file Table1.docx]

**Supplementary Table S1.** Multivariable negative binomial regression model for exacerbation frequency in patients with COPD (n = 96). Bold p-values indicate statistical significance (p < 0.05). BMI, body mass index; HOMA-IR, homeostatic model assessment of insulin resistance; OSAS, obstructive sleep apnoea syndrome; LTOT, long-term oxygen therapy; LABA, long-acting beta-agonist; LAMA, long-acting muscarinic antagonist; ICS, inhaled corticosteroid.

| **Predictor** | **IRR** | **Std. err.** | **P- value** | **95% CI** |
| --- | --- | --- | --- | --- |
| **Metabolic markers** |  | | | |
| HOMA-IR (log) | 1.14 | 0.13 | 0.265 | 0.91 – 1.42 |
| BMI (centred) | 1.01 | 0.01 | 0.676 | 0.98 – 1.03 |
| Leptin (log) | 0.84 | 0.18 | 0.405 | 0.56 – 1.27 |
| **Demographics** |  | | | |
| Age (years) | 1.01 | 0.01 | 0.311 | 0.99 – 1.02 |
| Male sex | 1.05 | 0.11 | 0.657 | 0.85 – 1.29 |
| Rural residence | 0.89 | 0.06 | 0.084 | 0.78 – 1.02 |
| **Comorbidities** |  | | | |
| Obesity | 1.17 | 0.16 | 0.234 | 0.90 – 1.53 |
| OSAS | 0.64 | 0.07 | **<0.001** | 0.53 – 0.79 |
| Type 2 diabetes | 1.01 | 0.11 | 0.961 | 0.82 – 1.23 |
| Hypertension | 0.73 | 0.05 | **<0.001** | 0.63 – 0.85 |
| Heart failure | 1.38 | 0.11 | **<0.001** | 1.18 – 1.62 |
| Atrial fibrillation | 0.92 | 0.08 | 0.333 | 0.78 – 1.09 |
| **Exposure & history** |  | | | |
|  |  | | | |
| Current smoking | 1.08 | 0.25 | 0.740 | 0.69 – 1.70 |
| Smoking duration (years) | 1.00 | 0.00 | 0.406 | 0.99 – 1.01 |
| Tuberculosis history | 1.05 | 0.11 | 0.609 | 0.86 – 1.28 |
| **Disease severity** |  | | | |
| COPD stage (GOLD) | 1.37 | 0.08 | **<0.001** | 1.22 – 1.55 |
| **Treatment regimen** |  | | | |
| LABA/LAMA (vs LABA) | 0.98 | 0.16 | 0.896 | 0.71 – 1.34 |
| ICS/LABA/LAMA (vs LABA) | 0.91 | 0.15 | 0.589 | 0.66 – 1.27 |
| LTOT | 1.03 | 0.09 | 0.749 | 0.87 – 1.22 |
